# Comparing single-site with multisite rTMS for the treatment of chronic tinnitus – clinical effects and neuroscientific insights: study protocol for a randomized controlled trial

**DOI:** 10.1186/1745-6215-14-269

**Published:** 2013-08-23

**Authors:** Astrid Lehner, Martin Schecklmann, Peter M Kreuzer, Timm B Poeppl, Rainer Rupprecht, Berthold Langguth

**Affiliations:** 1 Department of Psychiatry and Psychotherapy, University of Regensburg, Universitaetsstraße 84, Regensburg 93053, Germany

**Keywords:** Repetitive transcranial magnetic stimulation, Chronic tinnitus, Neuromodulation, Network

## Abstract

**Background:**

Several years ago, repetitive transcranial magnetic stimulation (rTMS) of the auditory cortex has been introduced as a treatment approach for chronic tinnitus. Even if this treatment is beneficial for a subgroup of patients, the overall effects are limited. This limitation may be due to the fact that the auditory cortex is only one of several brain areas involved in tinnitus. Whereas auditory areas are considered to code for tinnitus loudness, conscious perception of and attention allocation to tinnitus is supposed to be reflected by network activity involving frontal and parietal cortical areas. The aim of the present study is to influence this frontoparietal network more efficiently by perturbing the most important nodes with rTMS.

**Methods/design:**

This is a randomized, double-blind, parallel-group study. Patients receive rTMS treatment on 10 consecutive working days using either the multisite rTMS protocol (left dorsolateral prefrontal, 1,000 stimuli, 20 Hz; left temporoparietal, 1,000 stimuli, 1 Hz; right temporoparietal stimulation, 1,000 stimuli, 1 Hz) or a single-site protocol (unilateral stimulation of the temporoparietal cortex, 3,000 stimuli, 1 Hz). Individuals aged 18 to 70 years with chronic tinnitus ≥6-month duration and a Tinnitus Handicap Inventory score ≥38 are recruited for the study. A total of 50 patients are needed to detect a clinical relevant change of tinnitus severity (α = 0.05; 1 – β = 0.80). Primary outcome measures are the change in the Tinnitus Questionnaire score from baseline to the end of treatment as well as the number of treatment responders as defined by a reduction in the Tinnitus Questionnaire score of ≥5 points. Furthermore, changes in brain structure and activity are assessed using (functional) magnetic resonance imaging and electroencephalography in the resting state. Those measurements are also performed in 25 healthy control subjects.

**Discussion:**

This study is designed to reveal whether network stimulation is superior to single-site stimulation in the treatment of chronic tinnitus. Furthermore, the comparison between tinnitus patients and healthy controls and the longitudinal effects of both rTMS treatment protocols on brain structure and function allow inferences to be made about the neural correlates of tinnitus.

**Trial registration:**

Clinical Trials: NCT01663324

## Background

Subjective tinnitus is the auditory sensation of sound or noise in the absence of an acoustic stimulus. About 10 to 15% of adults in western societies report to suffer from tinnitus [1]. Tinnitus may have many etiologies and can involve severe psychological distress [2]. The neurobiological mechanisms underlying tinnitus are still incompletely understood, and causally oriented treatment options are scarce. Neuroimaging and electroencephalography (EEG)/magnetoencephalography (MEG) studies have revealed increased neural activity in central auditory pathways [3-8] and have demonstrated alterations in the frequency spectrum power during resting state activity in tinnitus patients [9]. Based on the hypothesis that repetitive transcranial magnetic stimulation (rTMS) can reduce tinnitus by interfering noninvasively with abnormal activity in the auditory cortex, this method has been investigated for the treatment of tinnitus [10-12]. rTMS treatment in tinnitus patients is so far still of questionable clinical benefit [13], and therefore is not yet ready for application in the clinical routine. Taking into account the lack of other causally oriented treatment options for tinnitus, rTMS treatment is nevertheless considered a promising technique, and miscellaneous optimization strategies have been proposed [14].

In the current study an innovative optimization approach is used that is motivated by the recent finding in tinnitus patients that alterations of brain activity are not restricted to the auditory cortex but involve a network of both auditory and nonauditory brain regions [15-17]. Important nodes of this tinnitus network include the left and right auditory cortex, the anterior and posterior cingulate cortex, left frontal and parietal cortical areas. These areas and their functional interconnection are thought to reflect the conscious perception of tinnitus and the attention allocation to the percept [17-20]. Aiming to increase the therapeutic efficacy of rTMS, we propose a new multisite stimulation protocol interfering with this tinnitus network at several nodes. The multisite stimulation protocol consists of high-frequency left dorsolateral prefrontal stimulation followed by left and right temporoparietal low-frequency rTMS and has already been tested in a pilot study [21]. This pilot study showed promising effects. The new protocol should now be examined in a double-blind randomized controlled trial (blinding of the patient and the clinicians involved in ratings and data analysis) to determine whether it is able to disrupt the altered activity within the tinnitus network. As tinnitus has been shown to be associated with altered connectivity patterns [15-17], EEG and functional magnetic resonance imaging (MRI) measurements will be used to replicate those results and to examine the effect of rTMS on these alterations.

### Objectives

Based on the findings in the pilot study, we hypothesize that the reduction of the Tinnitus Questionnaire (TQ) score as well as the number of treatment responders after 10 days of rTMS treatment and after follow-up periods of 3 and 6 months are higher when patients are treated using the new multisite stimulation protocol in comparison with single-site stimulation of the temporoparietal cortex. Treatment response is defined as an improvement of ≥5 points in the TQ score [22]. Further aims of the study are as follows: to compare multisite stimulation with single-site stimulation with respect to a change of tinnitus severity as measured by the TQ, the Tinnitus Handicap Inventory (THI) and numeric rating scales assessing the loudness and annoyance of the tinnitus percept (baseline vs. end of treatment and all follow-up visits); to compare single-site stimulation with multisite stimulation with respect to a change in secondary outcomes such as quality of life, hyperacusis and depressive symptoms (baseline vs. end of treatment and all follow-up visits); to replicate and expand findings concerning the neural correlates of tinnitus using resting state EEG, MRI and resting-state functional MRI; to examine whether successful treatment with rTMS is correlated with changes in brain structure and activity; to examine whether treatment with multisite rTMS induces different brain changes than treatment with single-site rTMS (we assume that network alterations associated with tinnitus can be influenced more effectively using multisite stimulation as compared with single-site rTMS); and to determine whether brain structure and activity as measured by EEG and functional MRI at baseline are predictive for treatment response.

## Methods/design

### Design of the trial

This trial is a two-arm, randomized, double-blind parallel-group study of 2 weeks of rTMS treatment plus 12-week and 24-week follow-up visits in patients with moderate to severe chronic subjective tinnitus. The trial is performed at the multidisciplinary tinnitus clinic at the University of Regensburg. Fifty patients are randomly assigned to a 2-week rTMS treatment either with a single-site rTMS protocol or with the new multisite protocol.

### Study population

#### Patients

Fifty patients are included in the study. Patients are eligible for study participation if they meet the following inclusion criteria: diagnosis of chronic subjective tinnitus; duration of tinnitus ≥6 months; score ≥38 on the THI at screening; age between 18 and 70 years; and written informed consent.

The study exclusion criteria were: objective tinnitus; treatable cause of the tinnitus; prior treatment with transcranial magnetic stimulation; clinically relevant unstable psychiatric, internal or neurological comorbidity; history or evidence of significant brain malformation, neoplasm, head injury or cerebral vascular events; metal objects in and around the body that cannot be removed, cardiac pacemakers, other electronic implants; history of seizures or epileptic activity; pregnancy; alcohol or drug abuse; and intake of benzodiazepines ≥1 mg lorazepam/day (or equivalent doses of other benzodiazepines).

### Control subjects

To potentially replicate and expand findings concerning the neural correlates of tinnitus, 25 control subjects without tinnitus but matched for age, gender and hearing function are examined once using EEG, MRI, functional MRI and all questionnaires (except for the tinnitus-specific ones). Inclusion criteria for the control subjects are age between 18 and 70 years and written informed consent. Exclusion criteria are identical to the exclusion criteria for patients except for the first three criteria.

### Procedures

**Figure 1:**
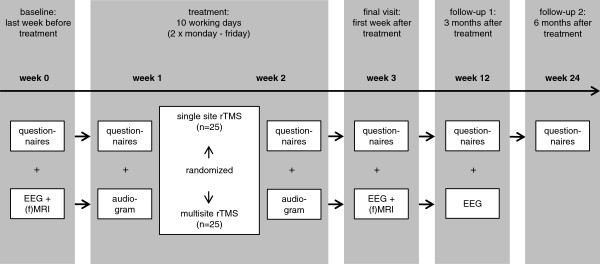
**Visit schedule.** EEG, electroencephalography; (f)MRI, (functional) magnetic resonance imaging; rTMS, repetitive transcranial magnetic stimulation.

Patients are recruited during routine clinical tinnitus consultations, via print-media announcement and via the homepage of the multidisciplinary tinnitus clinic at the University of Regensburg. At screening, all patients are asked to fill in the Tinnitus Sample Case History Questionnaire [23], and inclusion/exclusion criteria are checked. After screening and giving written informed consent, patients are examined in a baseline visit that involves structural MRI, resting state functional MRI and resting state EEG measurements (see Figure 1). Those measurements are repeated after completion of rTMS treatment. After a 3-month follow-up period, an additional resting state EEG measurement is done. Furthermore, questionnaires have to be filled out at different time points including follow-up visits after 3 and 6 months (see Figure 1).

### (Functional) MRI/electroencephalography

The MRI scans are performed on a Siemens Allegra 3-Tesla head scanner (Siemens AG, Erlangen, Germany) with a single channel headcoil. First, a high-resolution T1 weighted image (160 sagittal slices covering the whole brain, 1 × 1 × 1 mm^3^ voxel size, field of view = 256 × 256 mm^2^) is acquired from each subject using the ADNI sequence (repetition time = 2250 ms, echo time = 2.6 ms, flip angle = 9°). Then 6 minutes of resting state functional MRI are performed using a T2* weighted gradient echo-planar imaging sequence (repetition time = 2 seconds, echo time = 30 ms, 34 slices, field of view = 192 × 192 mm^2^, flip angle = 90°, 3 × 3 × 3 mm^3^ voxel size). The EEG equipment consists of the BrainAmp DC amplifier (Brain Products, Herrsching, Germany) and EEG recording caps with the possibility to place up to 64 electrodes (10–20 System; EasyCap, Gilching, Germany). The EEG is recorded from 62 electrodes with impedances below 10 kΩ and a sampling rate of 500 Hz. A resting state EEG measurement is carried out for 7 minutes with eyes closed.

### Questionnaires

To assess tinnitus severity, tinnitus patients have to fill in different tinnitus questionnaires at several time points (see Figure 1): the THI, the German version of the TQ [22,24] and numeric rating scales for tinnitus loudness and annoyance. In addition, depressive symptoms, quality of life and hyperacusis are assessed using the Major Depression Inventory, the World Health Organization Quality of Life assessment and a German hyperacusis questionnaire [25].

### Treatments

On the first treatment day, patients are randomized to one of the following two parallel groups.

The multisite group is treated with the new multisite rTMS protocol consisting of high-frequency stimulation of the left dorsolateral prefrontal cortex (20 Hz, 1000 stimuli/day, which are applied in 20 trains with an intertrain interval of 25 seconds) followed by 1000 stimuli at 1 Hz over the left temporoparietal cortex and 1000 stimuli at 1 Hz over the right temporoparietal cortex. The stimulation parameters have been chosen according to a pilot study [21] with a slight modification with respect to the number of stimuli. A detailed explanation for the choice of protocol can be found in Lehner and colleagues [21].

The single-site group is treated with a single-site rTMS protocol consisting of 3000 stimuli/day that are applied at 1 Hz over the temporoparietal cortex contralateral to the tinnitus percept [26]. Based on former studies, the left hemisphere is treated in case of bilateral tinnitus (for example [27]). Since the goal of this study is to demonstrate superiority of the multisite stimulation over the current standard approach (stimulation of the temporoparietal cortex), an active stimulation protocol was chosen for comparison instead of placebo stimulation.

All patients receive 10 treatment sessions on 10 consecutive working days. The total number of stimuli per session is identical for both treatment groups (3000 stimuli per session). rTMS is performed with an intensity of 110% of the resting motor threshold but never higher than 60% of the maximal stimulator output. The resting motor threshold is determined at the beginning of the first treatment session and is defined as the minimal intensity at which at least five of 10 motor evoked potentials are 50 μV in amplitude in the right abductor digiti minimi. rTMS treatment is performed with a Medtronic MagPro Option stimulator (Medtronic, Minneapolis, MN, USA) connected to a 70 mm figure-of-eight coil. The coil is held tangential to the scalp with the handle pointing upwards. The temporoparietal cortices are localized using the 10–20 System by placing the coil between the temporal (T3/T4) and the parietal (P3/P4) EEG electrode sites [28,29]. The dorsolateral prefrontal cortex is targeted by centering the coil 6 cm anterior from the part of the motor cortex that had been used for defining the resting motor threshold.

rTMS treatment is applied by nonblinded study staff not involved in any other study-specific procedures. Patient management and assessment, functional MRI and EEG measurements and the statistical analyses are done by psychologists and physicians who are blinded with respect to treatment conditions.

#### Outcome measures

The primary outcome measures are the change in the TQ score from baseline to end of treatment (interaction effect time × group) as well as the number of treatment responders as defined by a reduction in the tinnitus questionnaire score of ≥5 points at the end of treatment. Secondary outcome measures are changes in all scales and questionnaires (TQ, THI, numeric rating scales, Major Depression Inventory, World Health Organization Quality of Life assessment, hyperacusis questionnaire) between baseline and end of treatment as well as between baseline and all follow-up periods. Furthermore, the number of treatment responders at all follow-up time points is examined.

#### Blinding

Patients, clinical raters and data analysts are blind to treatment conditions. As the treatment group is easily realized by the number of brain areas stimulated (one in the control group vs. three in the treatment group), patients are informed that two active stimulation procedures are compared without specifying the exact difference between the two protocols.

#### Randomization

An online generator was used to prepare a balanced randomization list before the trial started. The whole sample was used as one block. Only the nonblinded clinical staff are granted access to this list and perform randomization prior to the first treatment session.

#### Determination of sample size

The study is designed to find an interaction effect between group (single-site vs. multisite) and time (baseline, end of treatment). Based on our pilot data [21] a small effect size of *f* = 0.1 for this interaction effect is assumed. Although small, such an effect is still an important step in tinnitus management. If the study sample size is determined to provide sufficient power (0.8) for detection of such an effect in a repeated-measures analysis of variance (with α = 0.05), a total of 42 tinnitus patients have to be examined. Due to the complex and time-consuming study design, a higher patient dropout rate than usual is assumed. A total of 50 patients (25 per group) are therefore planned to be included in the study.

#### Statistical analysis

Missing data entries are handled with the last observation carried forward method. For the comparison of treatment responders in both groups, a chi-square statistic is calculated. For all secondary outcomes, an analysis of variance is used with the between-subjects factor group (multisite rTMS vs. single-site rTMS) and the repeated-measures factor time (baseline vs. end of treatment vs. all follow-up time points). EEG and (functional) MRI data are analyzed with MatLab-based analytic software (MathWorks, Natick, MA, USA; EEGLAB; FieldTrip; SPM). For both EEG and functional MRI data, different exploratory analyses focusing predominantly on connectivity measures will be done. As it is hypothesized to find altered connectivity patterns in tinnitus patients, imaging/EEG data of both patient groups are compared with data for the control group (baseline measurements of the tinnitus groups vs. single measurement of the controls). Furthermore, longitudinal analyses will be performed to test for a change of the connectivity patterns from before to after rTMS treatment (contrast: pre rTMS vs. post rTMS). Correlations of imaging data and questionnaire scores are used to identify potential predictors for rTMS outcome. As the present protocol has a clinical focus, we do not go into detail concerning the EEG and functional MRI analyses at this point.

#### Ethical aspects

The study protocol has been approved by the ethics committee of the University of Regensburg (10-101-0169). The study is performed in agreement with the principles of the Declaration of Helsinki. All patients are informed about the purpose and the risks of the study and about their right to withdraw their consent at any time. Patients are only included in the study if written informed consent is given.

## Discussion

rTMS has been used as a treatment approach for chronic tinnitus for several years. Results from clinical trials are heterogeneous; in summary, the effect size of rTMS treatment over the auditory cortex has been shown to be rather small. The idea of interfering with altered brain activity via noninvasive brain stimulation is plausible, however, and the efficacy of a specific treatment protocol depends on the quality of the neurobiological model underlying that protocol. During the last few years, knowledge about the neurobiological mechanisms of tinnitus has advanced [20]. Neuroscientists have moved away from a model restricted to the auditory system towards the concept of a more widespread tinnitus network. This new model of tinnitus can and should now be used to improve rTMS treatment. The attempt to adapt rTMS treatment to the network model brings several methodological difficulties about which had to be dealt with in the design of this trial. The tinnitus network as described by Schlee and colleagues involves several brain regions, all of which can be stimulated with different frequencies in a different order and with a varying number of stimuli [17]. One of the many possible treatment protocols therefore had to be chosen [21]. The attempt of this study is not to find the best of all possible protocols but to determine whether a multisite stimulation protocol based on recent studies on the tinnitus network is superior to the standard protocol used in tinnitus treatment. This reason is also why the standard protocol was used as control treatment instead of a placebo control. Having these constraints in mind, the study is capable of providing useful information about a new way to use rTMS in tinnitus patients. By using EEG and (functional) MRI we are able to determine whether both treatment protocols induce different changes in brain structure and function and whether multisite stimulation serves the purpose of interfering with the tinnitus network. Furthermore, the comparison between clinical changes and changes in neuroimaging findings over time provides a unique opportunity for further specifying the exact role of the neuronal network changes underlying tinnitus perception and distress.

## Trial status

The trial started recruitment in July 2012 and will presumably complete follow-up assessments in 2014.

## Abbreviations

EEG: Electroencephalography; MRI: Magnetic resonance imaging; rTMS: Repetitive transcranial magnetic stimulation; THI: Tinnitus Handicap Inventory; TQ: Tinnitus Questionnaire.

## Competing interests

The authors declare that they have no competing interests.

## Authors’ contributions

AL, MS and BL designed the study and wrote the study protocol. BL is the principal investigator of the study. AL is the trial manager. AL, PMK, TBP and MS contribute to the recruitment of the patients and data collection. The statistical analysis plan has been developed by AL and MS. The manuscript has been drafted by AL. RR approved the study protocol. All authors read and approved the final manuscript.
