# Correction: comparing single-site with multisite rTMS for the treatment of chronic tinnitus - clinical effects and neuroscientific insights: study protocol for a randomized controlled trial

**DOI:** 10.1186/1745-6215-15-148

**Published:** 2014-04-27

**Authors:** Astrid Lehner, Martin Schecklmann, Peter M Kreuzer, Timm B Poeppl, Rainer Rupprecht, Berthold Langguth

**Affiliations:** 1 Department of Psychiatry and Psychotherapy, University of Regensburg, Universitaetsstraße 84, Regensburg, 93053, Germany

## Correction

Following publication of our article [1], we became aware that the treatment protocol for the single-site group is incorrectly stated in the methods section. All patients in this group are stimulated over the left temporoparietal cortex (not over the temporoparietal cortex contralateral to the tinnitus percept). Using the same stimulation site for all patients in the single-site group avoids the otherwise subsequently necessary division into (possibly dysbalanced) subgroups and hence ensures straightforward data analyses and interpretability, especially with respect to the longitudinal imaging data.

## References

[B1] Lehner A Schecklmann M Kreuzer PM Poeppl TB Rupprecht R Langguth B Comparing single-site with multisite rTMS for the treatment of chronic tinnitus - clinical effects and neuroscientific insights: study protocol for a randomized controlled trial Trials 2013 14 269 10.1186/1745-6215-14-269 23968498 PMC3765439

